# Modeling peak ground acceleration for earthquake hazard safety evaluation

**DOI:** 10.1038/s41598-024-82171-7

**Published:** 2024-12-28

**Authors:** Fatima Khalid, Milad Razbin

**Affiliations:** 1 Department of Civil Engineering, NED University of Engineering and Technology, Karachi, Pakistan; 2 Department of Textile Engineering, Amirkabir University of Technology, Tehran, Iran

**Keywords:** Earthquake, Peak ground acceleration, Artificial neural network, Genetic algorithm, Natural hazards, Civil engineering, Computational science

## Abstract

This paper presents a ground motion prediction (GMP) model using an artificial neural network (ANN) for shallow earthquakes, aimed at improving earthquake hazard safety evaluation. The proposed model leverages essential input variables such as moment magnitude, fault type, epicentral distance, and soil type, with the output variable being peak ground acceleration (PGA) at 5% damping. To develop this model, 885 data pairs were obtained from the Pacific Engineering Research Center, providing a robust dataset for training and validation. The ANN architecture comprises 4 nodes in the input layer, two hidden layers each containing 25 nodes, and a single-node output layer, resulting in 750 unknown weight and bias values that the model must optimize. Following the model assessment, a genetic algorithm (GA) was integrated with the ANN model to enhance its predictive capabilities. This integration aimed to forecast 20 potential earthquake scenarios, a crucial step in validating the model’s effectiveness. The results were promising, as the ANN-GA successfully predicted earthquake occurrences in 15 out of 20 scenarios. These findings underscore the model’s potential in accurately forecasting seismic events, thereby contributing to the development of more resilient infrastructure and better-informed urban planning strategies.

## Introduction

Ground motion prediction equations (GMPEs) calculate the intensity measures of ground movements, essential for structural design and seismic risk evaluation. Several techniques and parameters, such as response spectrum, frequency content measures, earthquake magnitude, epicentral distance, fault type, soil type, and site classification parameters, have been adopted for developing GMPEs. The significant parameter usually predicted by GMPE is peak ground acceleration (PGA). In the study of PGA components, horizontal movements are deemed more critical for engineering than vertical ones, prompting most research to focus on horizontal aspects^1–4^. Typically, three approaches are used: first, the larger of the two horizontal components is selected; second, both horizontal components are considered; and third, the geometric mean of the estimates from both components is computed.

Mechanistic and empirical models, such as stochastic models and attenuation relations, have been developed, resulting in numerous GMPEs. From 1964 to 2021, 485 GMPEs for PGA and 316 for spectral ordinates were developed^5^. The accuracy of predictions relies on both the quality and quantity of data used to estimate the parameters. However, beyond the data itself, a key factor in inferring a GMPE is choosing the most suitable functional form. Initially, Esteva and Rosenblueth proposed a GMPE to predict PGA using a simple exponential function^6^. Several models based on synthetic data were proposed, but with advancements, models incorporating the effects of amplification, attenuation, fault mechanisms, etc., became more complex. Boore and Joyner presented strong ground motion prediction equations developed from earthquakes in California and Italy using peak acceleration as the most common measure, utilizing regression methods. The data was extracted from the updated NGA-West 2 database^7^. Bommer et al.. introduced an empirical approach to deriving GMPEs for estimating short-period response spectral ordinates across Europe and the Middle East, covering a magnitude range of 3.0 to 7.6^8^. Boore and Atkinson formulated a GMPE with dependent variables include PGA, PGV, and 5% PSA. Notably, the horizontal components derived are not simple geometric means but are calculated using the 50th percentile values from all possible orientations^3^. Campbell and Bozorgnia developed an empirical ground motion model that estimates PGA, PGV, PGD, and 5% damped linear elastic response spectra for time periods between 0.01 and 10 s. It explicitly incorporates effects such as magnitude saturation, attenuation dependent on magnitude, style of faulting, rupture depth, hanging-wall geometry, and site response, both linear and nonlinear. The model’s results suggest that ground motion predictions were fairly consistent across different distances, with significantly increased readings during reverse-faulting scenarios where ruptures don’t reach the surface. Higher ground motions were also observed in some strike-slip ruptures with limited surface expression, though other strike-slip cases showed divergent results. The model’s reliability may decrease near the extremes of its magnitude range^9^. Ansari et al. developed advanced models for predicting tunnel damage in seismically active and landslide-prone regions, using Artificial Neural Networking (ANN) and deep learning techniques. The multi-hazard damage prediction model and the seismic tunnel damage prediction model both employ feed-forward neural networks trained with critical input parameters, such as PGA, source-to-site distance, overburden depth, tunnel depth, lining thickness, and geological strength index. Validated against significant seismic events like the Kobe (1995), Chi-Chi (1999), Mid-Niigata (2004), and Wenchuan (2008) earthquakes, these models demonstrated high predictive accuracy and reliability, providing crucial seismic design recommendations to enhance post-disaster infrastructure serviceability. However, limitations include the uneven distribution of seismic data and constraints on model generalizability across varied geological conditions, necessitating further validation for broader application. The research outputs also feature damage indexing, predictive graphs, and globally applicable design guidelines^10,11^. In the following years, the structure of GMPEs was adjusted several times to account for factors such as amplification, attenuation, faulting mechanisms, damping, and the uncertainties observed in ground motions. These changes resulted in highly intricate functional models. A significant limitation of this type of parametric model is the need for a predefined functional structure.

The essential need for reliable earthquake prediction has long been a subject of debate, challenging even the most esteemed scientists. Precisely predicting the exact time, location, and magnitude of an earthquake is exceedingly complex due to the multitude of seismic precursors and other indicators of potential quakes. However, advancements in artificial intelligence (AI) and the aggregation of data from these indicators offer a promising avenue for improving earthquake prediction accuracy^12,13^. The utilization of advanced technological models like machine learning algorithms, ANN, and fuzzy logic has become increasingly prevalent in the field of earthquake engineering to interpret trends and yield accurate outcomes. Furthermore, AI enhances computational efficiency and reduces error rates, offering significant advantages in tasks such as phase picking, early warning systems, ground-motion prediction, tomography, and geodesy. Herein, a ground motion prediction model utilizing an ANN specifically for shallow earthquakes, with the goal of enhancing earthquake hazard safety evaluation, is introduced. The model incorporates key input variables, including moment magnitude, fault type, epicentral distance, and soil type, and predicts PGA at 5% damping. After assessing the model, a genetic algorithm (GA) was integrated with the ANN model. This combination aimed to predict potential earthquake scenarios.

## Modeling peak ground acceleration

**Fig. 1:**
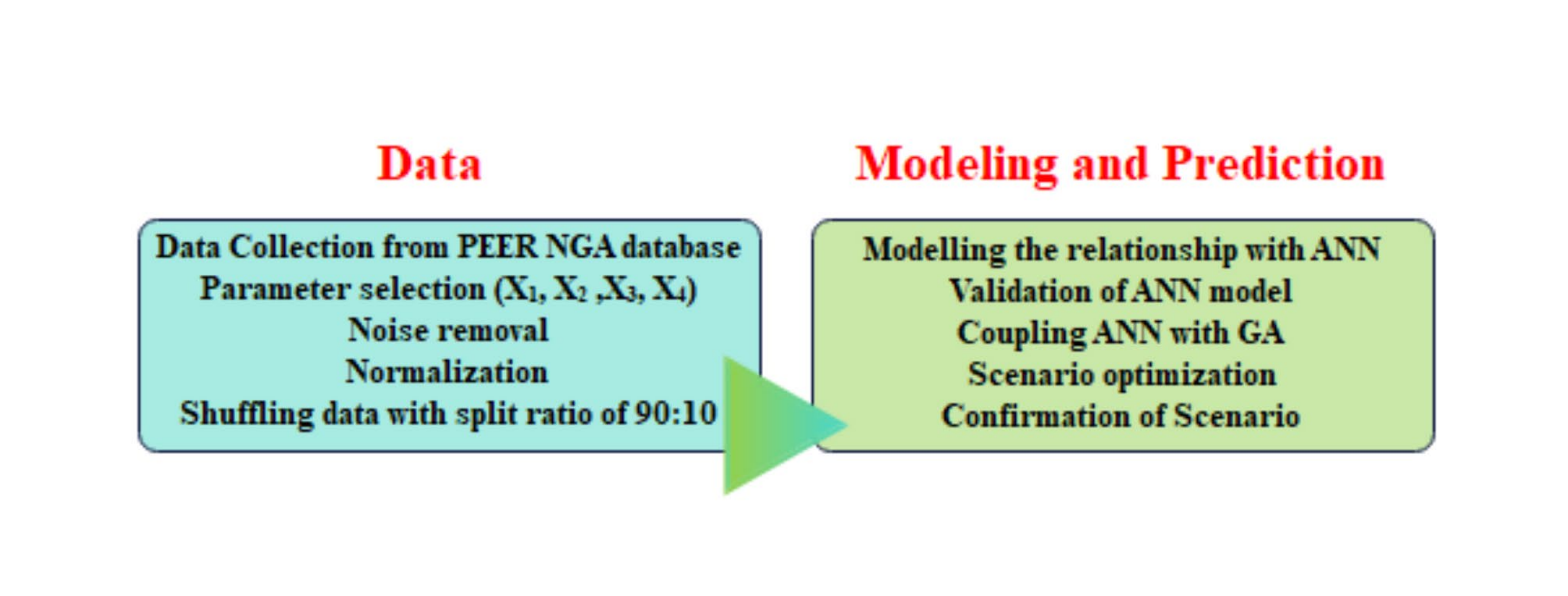
Work flow of the study to predict PGA at 5% damping using ANN-GA.

Before explaining the modeling of PGA in detail, an overview of the methodologies adopted in this study is shown in Fig. 1. The entire study consists of two main stages: data processing and modeling for prediction. In the following sections, more details will be provided for each stage.

### Data acquisition and preprocessing of PGA

The data was sourced from the Pacific Earthquake Engineering Research Center (PEER). This database encompasses a comprehensive collection of ground motions recorded during shallow earthquakes in active tectonic areas around the world. For this study, data from 100 unscaled original earthquake response spectra were gathered, specifically focusing on no-pulse records that do not consider the effects of velocity pulses due to rupture directivity. The input variables for the study include moment magnitude, fault type, epicentral distance, and soil type, with the output variable being PGA. Based on PEER, fault types are categorized by rake angle as summarized in Table 1. Meanwhile, soil type is grouped according to the National Earthquake Hazards Reduction Program (NEHRP) standards, utilizing (time-averaged shear-wave velocity to a depth of 30 m) Vs_30_ values as summarized in Table 2.

**Table 1:** Fault mechanism according to rake angle.

Fault Mechanism	Rake angle ($\:^\circ\:$)
Strike slip	− 180 < Rake < − 15 & − 30 < Rake < 30 & 150 < Rake < 180
Normal	− 120 < Rake < − 60
Reverse	60 < Rake < 120
Reverse oblique	30 < Rake < 60 & 120 < Rake < 150
Normal oblique	− 150 < Rake < − 120 & − 60 < Rake < − 30

**Table 2:** Soil type classification on basis of Vs_30_.

Soil type	V_s30_ (m/s)	Properties
A	> 1500	Hard rock
B	760–1500	Rock
C	360– 760	Very dense soil and soft rock
D	180– 360	Stiff soil profile
E	< 180	Soft soil profile

In this study, ground motion data were collected from recordings featuring earthquake magnitudes between 5.0 and 8.0 and epicentral distances not exceeding 200 km. Any records lacking complete information were omitted from the dataset. After these adjustments, the study comprised a total of 883 ground motions from 89 events, as detailed in Table 3.

**Table 3:** Earthquake events used in analysis.

Earthquake	X_1_	Mechanism	Earthquake	X_1_	Mechanism
Anza (Horse Canyon)	5.19	Strike Slip	Little Skull Mtn, NV	5.65	Normal
Baja California	5.50	Strike Slip	Livermore-01	5.80	Strike Slip
Big Bear-01	6.46	Strike Slip	Livermore-02	5.42	Strike Slip
Bishop (Rnd Val)	5.82	Strike Slip	Loma Prieta	6.93	Reverse-Oblique
Borah Peak, ID-01	6.88	Normal	Lytle Creek	5.33	Reverse-Oblique
Borah Peak, ID-02	5.10	Normal	Mammoth Lakes-01	6.06	Normal-Oblique
Borrego Mtn	6.63	Strike Slip	Mammoth Lakes-02	5.69	Strike Slip
CA/Baja Border Area	5.31	Strike Slip	Mammoth Lakes-03	5.91	Strike Slip
Caldiran, Turkey	7.21	Strike Slip	Mammoth Lakes-04	5.70	Strike Slip
Cape Mendocino	7.01	Reverse	Mammoth Lakes-06	5.94	Strike Slip
Chalfant Valley-01	5.77	Strike Slip	Mammoth Lakes-10	5.34	Strike Slip
Chalfant Valley-02	6.19	Strike Slip	Mammoth Lakes-11	5.31	Strike Slip
Chalfant Valley-04	5.44	Strike Slip	Managua, Nicaragua 01	6.24	Strike Slip
Chi-Chi, Taiwan	7.62	Reverse-Oblique	Manjil, Iran	7.37	Strike Slip
Chi-Chi, Taiwan-02	5.90	Reverse	Mohawk Val, Portola	5.17	Strike Slip
Chi-Chi, Taiwan-03	6.20	Reverse	Morgan Hill	6.19	Strike Slip
Chi-Chi, Taiwan-04	6.20	Strike Slip	Mt. Lewis	5.60	Strike Slip
Chi-Chi, Taiwan-05	6.20	Reverse	N. Palm Springs	6.06	Reverse-Oblique
Chi-Chi, Taiwan-06	6.30	Reverse	Nahanni, Canada	6.76	Reverse
Coalinga-01	6.36	Reverse	Nenana Mt., Alaska	6.70	Strike Slip
Coalinga-02	5.09	Reverse	New Zealand-02	6.60	Normal
Coalinga-03	5.38	Reverse	Norcia, Italy	5.90	Normal
Coalinga-04	5.18	Reverse	Northridge-01	6.69	Reverse
Coalinga-05	5.77	Reverse	Northridge-04	5.93	Reverse-Oblique
Coalinga-07	5.21	Reverse	Northridge-05	5.13	Reverse-Oblique
Coalinga-08	5.23	Strike Slip	Northridge-06	5.28	Reverse
Coyote Lake	5.74	Strike Slip	Oroville-01	5.89	Normal
Denali, Alaska	7.90	Strike Slip	Parkfield	6.19	Strike Slip
Dinar, Turkey	6.40	Normal	San Fernando	6.61	Reverse
Double Springs	5.90	Strike Slip	San Francisco	5.28	Reverse
Duzce, Turkey	7.14	Strike Slip	San Juan Bautista	5.17	Strike Slip
Friuli, Italy-01	6.50	Reverse	Santa Barbara	5.92	Reverse-Oblique
Friuli, Italy-02	5.91	Reverse	Sierra Madre	5.61	Reverse
Gazli, USSR	6.80	Strike Slip	Sitka, Alaska	7.68	Strike Slip
Gulf of California	5.70	Strike Slip	Spitak, Armenia	6.77	Reverse-Oblique
Hector Mine	7.13	Strike Slip	Superstition Hills-01	6.22	Strike Slip
Hollister-04	5.45	Strike Slip	Superstition Hills-02	6.54	Strike Slip
Imperial Valley-06	6.53	Strike Slip	Tabas, Iran	7.35	Reverse
Imperial Valley-07	5.01	Strike Slip	Upland	5.63	Strike Slip
Irpinia, Italy-01	6.90	Normal	Victoria, Mexico	6.33	Strike Slip
Irpinia, Italy-02	6.20	Normal	Westmorland	5.90	Strike Slip
Kern County	7.36	Reverse	Whittier Narrows-01	5.99	Reverse-Oblique
Kobe, Japan	6.90	Strike Slip	Whittier Narrows-02	5.27	Reverse-Oblique
Kocaeli, Turkey	7.51	Strike Slip	Yountville	5.00	Strike Slip
Landers	7.28	Strike Slip			

The statistical properties of the experimental data are summarized in Table 4. Each input parameter was coded as X_1_, X_2_, X_3_, and X_4_, corresponding to moment magnitude, fault type, epicentral distance, and soil type, respectively. The fault mechanism was assigned a number from 0 to 4, and the soil type was given a value from 1 to 4, representing soil types B to E, as shown in Table 2. No data were recorded for soil type A.

**Table 4:** Descriptive statistics of the data used in this research.

Parameter	Moment magnitude	Fault type	Epicentral distance (km)	Soil type	PGA
Code	X_1_	X_2_	X_3_	X_4_	Y
Upper limit	5.0000	0.0000	0.4400	1.0000	0.0054
Bottom limit	7.9000	4.0000	198.9700	4.0000	0.6221
Range	2.9000	4.0000	198.5300	3.0000	0.6167
Average	6.2363	1.2971	57.9128	2.5510	0.1330
Variance	0.5157	1.4440	1742.1141	0.3635	0.0072
Skewness	0.1230	0.0978	1.0101	-0.3991	1.5625
Median	6.2000	2.0000	50.0050	3.0000	0.0959
Kurtosis	-0.6635	-1.5566	0.7526	-0.2349	2.3725

**Fig. 2:**
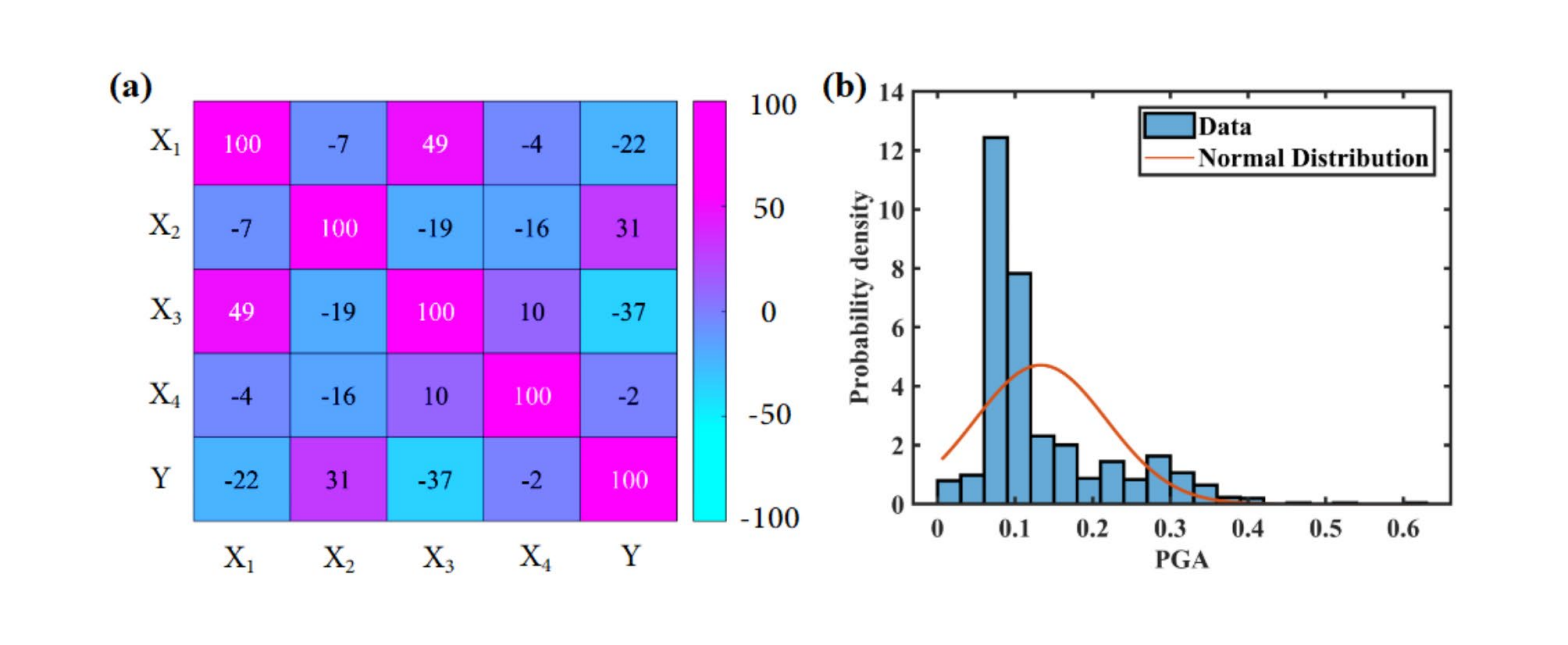
(**a**) Pearson’s correlation heatmap matrix of data space and (**b**) data distribution of Y.

In this study, we conducted a comprehensive analysis of the dataset, including variables X_1_, X_2_, X_3_, and X_4_, by examining their pairwise associations through a Pearson correlation matrix. The results, shown in Fig. 2a, reveal that there is no substantial linear correlation among these variables. All correlation coefficients are either close to zero or fall significantly below the threshold for moderate correlation (< 90%), indicating a lack of strong linear relationships. These findings highlight the relative independence of the variables, suggesting that linear modeling techniques may not be suitable for capturing their interdependencies. In Fig. 2b the plot is comparing the distribution of PGA values with a normal distribution curve. The data appears to be skewed, with a large concentration of values at lower PGA levels, deviating from the normal distribution’s symmetry suggesting that the PGA data is not normally distributed.

To mitigate quantitative effect of features, the datasets were subjected to a normalization process ranging between ($\:a=$ 0.1) and ($\:b=$ 0.9) prior to training the model, as given in Eq. (1)^14^.

1 $$\:{x}_{n}=\left(b-a\right)\left(\frac{x-{x}_{min}}{{x}_{max}-{x}_{min}}\right)+a$$

### Modeling and prediction of PGA

An ANN is a computational framework inspired by the neural architecture of the human brain. It consists of interconnected units known as neurons, which are organized into various layers such as an input layer, several hidden layers, and an output layer. Each neuron in these layers is linked through weights that influence the signals transmitted between neurons. ANNs are employed in machine learning for their ability to adaptively learn from data, enabling them to perform tasks such as pattern recognition, classification, and regression^15^. For an ANN with two hidden layers, the final output $\:y$ can be expressed as:

2 $$\:y=f\left(\sum\:_{k=1}^{{m}_{2}}{w}_{k}^{\left(3\right)}f\left(\sum\:_{j=1}^{{m}_{1}}{w}_{jk}^{\left(2\right)}f\left(\sum\:_{j=1}^{n}{w}_{ij}^{\left(1\right)}{x}_{i}+{b}_{j}^{\left(1\right)}\right)+{b}_{k}^{\left(2\right)}\right)+{b}^{\left(3\right)}\right)$$

Where $\:x$ is Input features, $\:w$ is weights matrix, $\:b$ is biases values and $\:f$ is activation function. $\:{m}_{1}$, $\:{m}_{2}$, and $\:{m}_{3}$ are number of neurons in input, first hidden layer and second hidden layer, respectively^16^. In this study, an ANN model with two hidden layers utilizing the settings summarized in Table 5 under MATLAB software was developed to map four input features to a single output.

**Table 5:** Value of hyperparameter settings for ANN.

Parameter	Value
Normalization domain	[0.1,0.9]
Data split ratio of training to testing data	90:10
Units in input layer	4
Units in first hidden layer	25
Units in second hidden layer	25
Units in output layer	1
Activation function of first hidden layer	Hyperbolic tangent sigmoid
Activation function of second hidden layer	Hyperbolic tangent sigmoid
Activation function of output layer	Pure linear
Learning algorithm	Levenberg–Marquardt algorithm
Learning rate	0.90
Momentum value	0.90
Number of epochs	875
Number of runs	100
Network type	Feed-forward back-propagation
Software	MATLAB

During the training and testing steps, the performance of ANN models with different nodes in the hidden layers was evaluated using the total goodness function (TGF), as utilized by several authors^17–19^, as given in Eq. (3). This evaluation is crucial because it allows for the selection of the best weight and bias values for the ANN model.

3 $$\:TGF=\frac{1}{N}\sum\:_{i=1}^{2}{{n}_{i}({R}^{2}+1/{e}^{MSE})}_{i}$$

In which

4 $$\:MSE=\frac{1}{n}\sum\:_{i=1}^{n}{\left[{a}_{i}-{O}_{i}\right]}^{2}$$

5 $$\:{R}^{2}=1-\frac{\sum\:_{i=1}^{n}{\left({a}_{i}-{O}_{i}\right)}^{2}}{\sum\:_{i=1}^{n}{\left({a}_{i}-\stackrel{-}{a}\right)}^{2}}$$

Where $\:{a}_{i}$, $\:{O}_{i}$, $\:\stackrel{-}{a}$, n and $\:N$ are the target, output, mean values of the target, number of data during the testing or training step of the network and total number of data, respectively. Following the modeling, a GA, originally developed by John Holland^20^, was utilized in conjunction with an ANN to predict PGA. To simulate earthquake scenarios, a cost function based on Euclidean distance was determined as follows:

6 $$\:For\:function\:f:\:{R}^{4}⟶R,\:find\:\:\overrightarrow{x}\:\in\:\:{R}^{4}\:such\:that\:\sqrt{{f}_{n}\left(\overrightarrow{x}\right)-1.7016}=0\:\:\forall\:\:x\in\:\:{R}^{4}$$

7 $$\:{\overrightarrow{x}}_{i}=\left\{{{X}_{1}}_{i},{{X}_{2}}_{i},{{X}_{3}}_{i},{{X}_{4}}_{i}\right\}\:\:\&\:\:\:i=1.\dots\:.n$$

Where $\:\overrightarrow{x}$ is a vectors that stores input parameters, and $\:{X}_{1}$, $\:{X}_{2}$, $\:{X}_{3}$, and $\:{X}_{4}$ are the input parameters. The hyperparameter settings of the GA under MATLAB software are summarized in Table 6. The population consists of 25 individuals with a double vector representation, and the algorithm runs for a maximum of 25 generations. A uniform creation function initializes the population, with rank scaling and roulette selection enhancing diversity and selection pressure. One elite individual is carried over to the next generation. The mutation function uses adaptive feasible strategies, applied to 50% of the population, while a two-point crossover method is employed with a crossover fraction of 50%. Migration occurs forward at 10% intervals, with 90% of the population migrating. A penalty-based approach addresses nonlinear constraints, and the cost limit is set to zero.

**Table 6:** Value of hyperparameter settings for GA.

Parameter	Value
Population size	25
Population type	Double vector
Max generation	25
Creation function	Uniform
Scaling function	Rank
Selection function	Roulette
Elite count	1
Mutation function	Adapt feasible
Mutation fraction	0.50
Crossover function	Two points
Crossover fraction	0.50
Migration direction/ interval	Forward/ 10% of population size
Migration fraction	0.90
Nonlinear constraints algorithm	Penalty
Cost limit	0

## Results and discussion

**Fig. 3:**
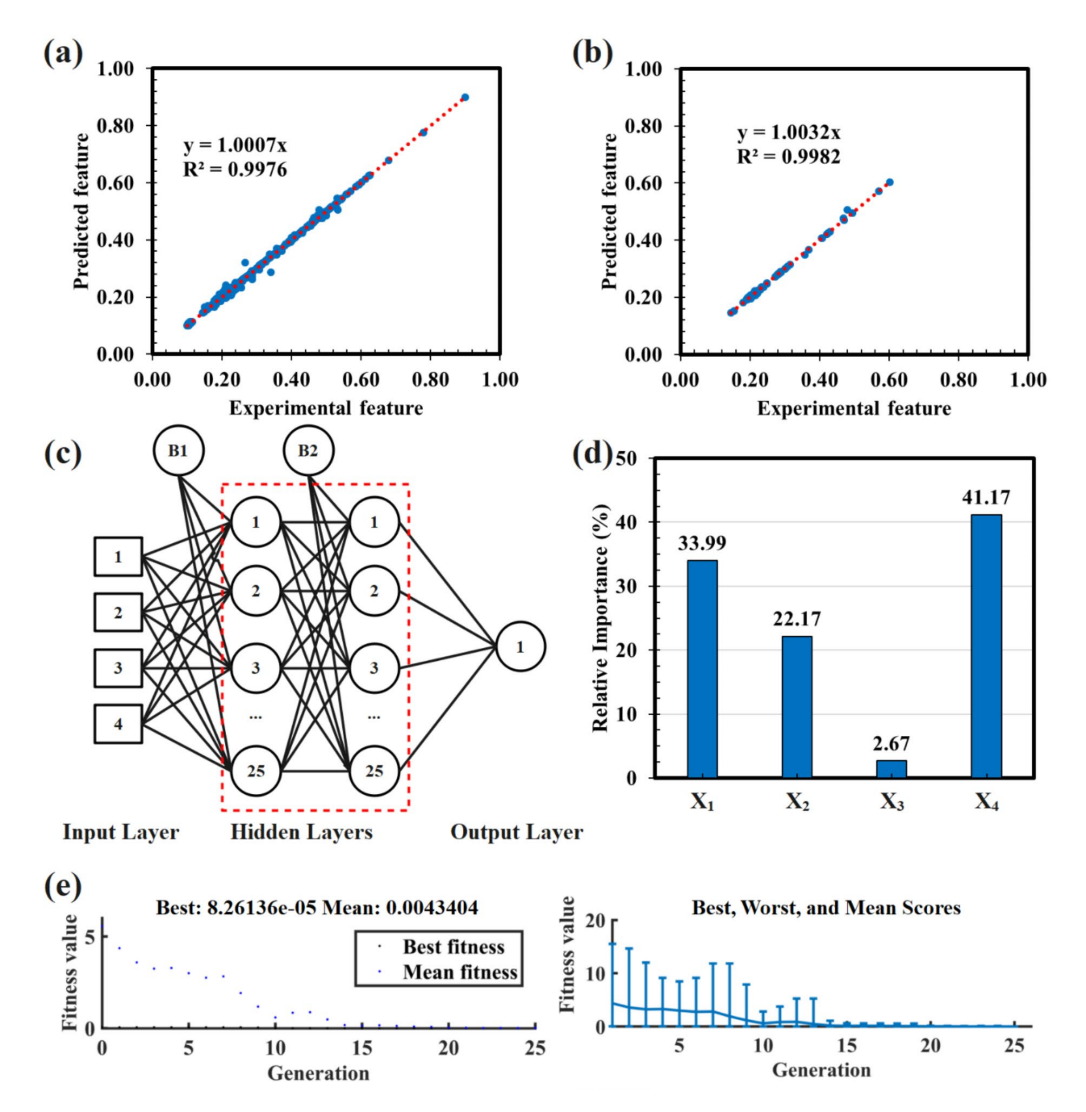
Performance of developed network during (**a**) training and (**b**) testing steps, (**c**) topology of developed network, (**d**) relative importance of different independent parameters, and (e) solving performance of genetic algorithm.

Figure 3a-b displays the performance of the ANN-based models during both the training and testing phases. The results indicate that the developed networks perform exceptionally well in both stages, as evidenced by the impressive R² values. Specifically, the training phase achieved an R² value of 0.9976, while the testing phase reached 0.9982. These high R² values demonstrate the excellent predictive capability of the ANN-based models.

The ANN architecture described in Fig. 3c features two hidden layers, each with 25 neurons, resulting in a configuration of 4-25-25-1. This specific setup has been identified as optimal for achieving the highest total goodness value. Figure 3d illustrates the relative importance of each input variable, revealing that soil type and epicentral distance contribute the most (41.17%) and the least (2.67%), respectively, to the output of the developed model.

To solve the problem, at the start of the optimization process, the GA assesses each individual by their cost values, targeting the individual with the minimum cost relative to the set input variables. The efficacy of the GA during the optimization is presented in Fig. 3e. It is important to highlight that the best result from the optimization is the average of 15 simulation runs, reflecting the GA’s inherent randomness. This approach ensures the robustness and reliability of the optimization results, taking into account the stochastic nature of the GA process.

Overall, the combined use of ANN and GA in this study has proven to be highly effective in predicting ground motions, providing valuable insights for seismic hazard assessments and earthquake engineering. The integration of these advanced computational techniques offers a powerful tool for improving the safety and resilience of structures in earthquake-prone areas.

**Fig. 4:**
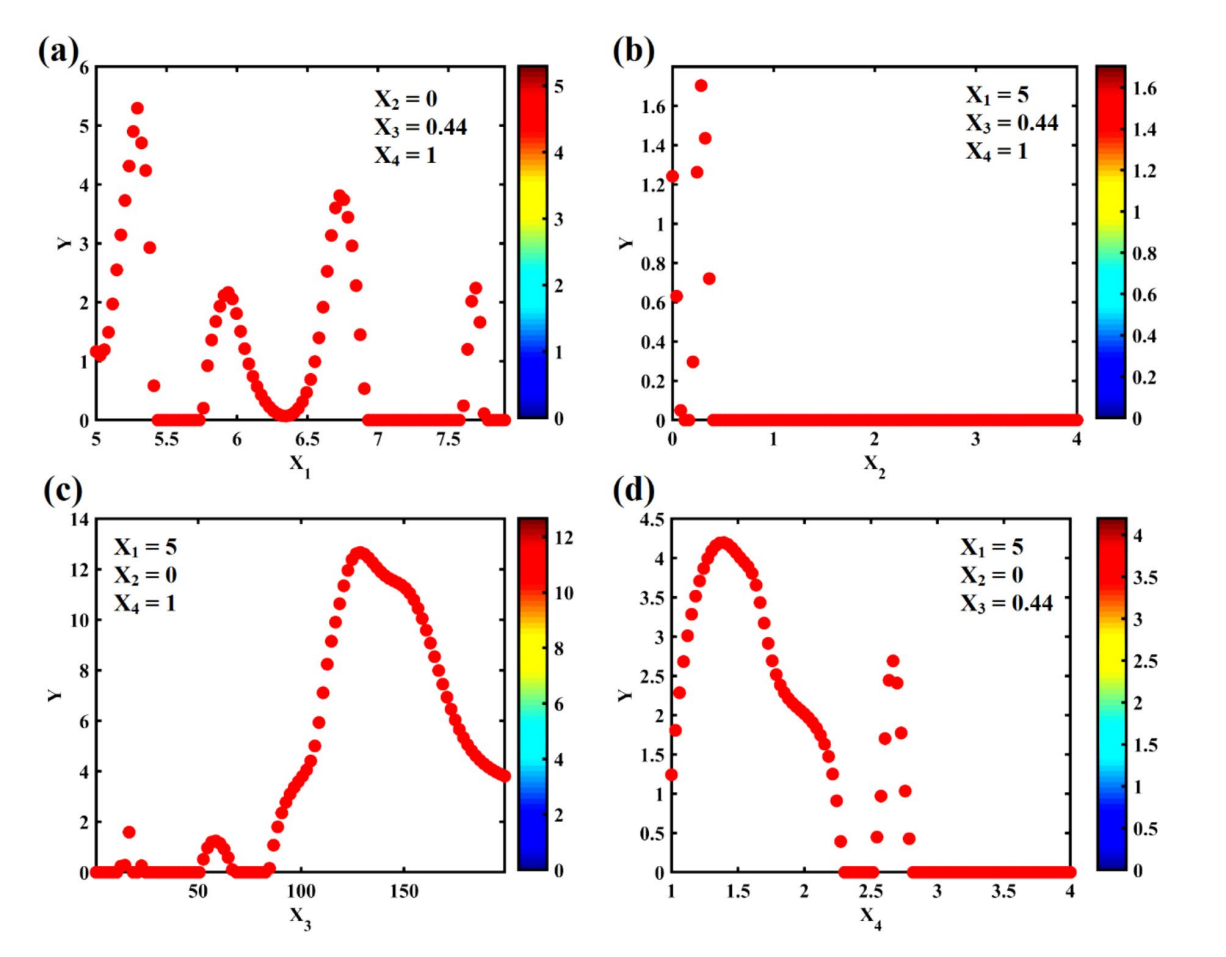
Effect of four key seismic factors: (**a**) moment magnitude (X_1_), (**b**) fault type (X_2_), (**c**) epicentral distance (X_3_), and (**d**) soil type (X_4_) on peak ground acceleration (Y).

Figure 4 illustrate the relationships between Peak Ground Acceleration (PGA) and four key seismic factors: moment magnitude (X_1_), fault type (X_2_), epicentral distance (X_3_), and soil type (X_4_). Each plot reveals the unique and complex influence of these factors on PGA. All these plots are obtained for a scenario with a moment magnitude of 5, fault type of 0, epicentral distance of 0.44, and soil type of 1. Starting with moment magnitude, we observe a strong non-linear correlation where larger magnitudes correspond to increased PGA values, but the relationship also contains peaks and troughs, indicating that other factors may modulate the effect. Fault type, while showing variation in PGA, has a relatively lower and more limited impact, suggesting that, for this case, fault type does not significantly affect ground acceleration compared to other parameters. Epicentral distance shows a clear inverse relationship with PGA—closer distances to the epicenter result in higher PGA, a physically intuitive outcome since seismic waves lose energy as they travel farther. Soil type, however, exhibits a non-linear effect, where certain soil types significantly amplify the ground acceleration, reflecting the well-known influence of soil characteristics on seismic wave behavior.

For this specific case, among these factors, moment magnitude and epicentral distance stand out as the most influential in determining PGA. Moment magnitude drives the energy release of an earthquake, directly affecting ground acceleration, while epicentral distance dictates how much energy reaches a specific location. Soil type plays a critical, albeit secondary, role, especially in areas prone to amplification due to soft soils. For the case under consideration, moment magnitude emerges as the most important factor, as it fundamentally governs the energy produced during an earthquake, which in turn influences all subsequent seismic behavior, including PGA.

Table 7 presents the outcomes for a set of 20 distinct fault combinations archived by ANN-GA system. It is well-established that reverse faults possess the capacity to yield elevated PGAs, especially in cases characterized by substantial fault slip and proximity to the Earth’s surface. In contrast, PGAs associated with normal oblique faults typically exhibit lower values compared to those emanating from reverse and strike-slip faults which was consistent with the results of^21–23^. Existing literature suggests that given the same earthquake magnitude, distance to the site, and site condition, the ground motions from normal-faulting earthquakes tend to be smaller than those from strike-slip earthquakes by about 20%. Upon careful examination of the data, a notable trend becomes apparent: for reverse and reverse-oblique faults (with X_2_ values of 2 and 3), even when the earthquake magnitude is substantial, the optimized PGA values are observed at greater epicentral distances. This observation suggests that these fault types are capable of generating heightened PGAs even when located at more substantial distances from the epicenter. In the case of normal faults (with an X_2_ value of 1), our analysis indicates that the maximum PGA is most prominent at shorter epicentral distances, with their impact diminishing as distances increase. However, for normal-oblique faults (with an X_2_ value of 4), the obtained results appear inconsistent with the underlying physical principles of the problem. Given that, in the context of normal-oblique faults, the PGA values at larger epicentral distances are typically negligible according to existing knowledge, which suggest higher PGAs at greater distances, raise questions regarding the validity of the optimization procedure. Further investigation and validation may be warranted to reconcile this discrepancy between the model results and the known physics of normal-oblique fault behavior. It is to be noted fault parameter uncertainty including fault location, slip rate, rupture mechanism, and fault geometry is highly crucial in predictions.

**Table 7:** Predicted scenarios for shallow earthquakes.

*N*	X_1_	X_2_	X_3_	X_4_	Cost value	Confirmation
1	5.8109	0.0000	58.6837	1.0000	8.26E−05	Yes
2	5.8229	0.0000	194.1308	2.0000	3.88E−04	Yes
3	6.7589	0.0000	111.1949	3.0000	1.91E−05	Yes
4	5.9791	0.0000	37.8381	4.0000	5.70E−04	Yes
5	6.4348	1.0000	9.5724	1.0000	8.11E−05	Yes
6	6.6769	1.0000	28.7305	2.0000	6.30E−04	Yes
7	5.4644	1.0000	54.8869	3.0000	8.31E−05	Yes
8	5.7881	1.0000	151.6950	4.0000	8.42E−04	No
9	5.9940	2.0000	62.4558	1.0000	1.87E−04	Yes
10	7.5194	2.0000	51.6856	2.0000	5.85E−05	Yes
11	7.0539	2.0000	34.8850	3.0000	5.36E−06	Yes
12	7.2667	2.0000	191.3018	4.0000	1.02E−04	Yes
13	6.8600	3.0000	173.9800	1.0000	9.50E−05	Yes
14	5.9092	3.0000	92.3098	2.0000	6.55E−04	Yes
15	6.8372	3.0000	152.9855	3.0000	8.95E−05	Yes
16	5.7547	3.0000	56.1277	4.0000	4.52E−04	Yes
17	7.3414	4.0000	124.1986	1.0000	6.07E−04	No
18	6.3177	4.0000	131.2216	2.0000	2.95E−05	No
19	6.5762	4.0000	107.7455	3.0000	9.51E−05	No
20	6.5348	4.0000	53.7205	4.0000	1.17E−04	No

The calibration of ANN-based models for region-specific seismic data is a critical step in ensuring accurate and reliable predictions of Peak Ground Acceleration (PGA). The data selected is of high-quality and it captures localized features such as Vs30, fault mechanisms, and attenuation characteristics for region-specific seismic prediction. Furthermore, model robustness was enhanced through hyperparameter tuning, cross-validation, and the application of regularization techniques to mitigate overfitting. These measures ensure that the ANN can generalize effectively to both training data and unseen events, contributing to the broader generalizability of PGA predictions. Incorporating diverse and representative datasets further strengthens the model’s adaptability to varying seismic conditions, making it suitable for practical seismic hazard evaluations.

Moreover, the analysis of the seismic data highlights a distinct pattern in PGA related to both epicentral distance and moment magnitude. The findings indicate a pronounced reduction in PGA as the epicentral distance increases, supporting the inverse square law. This law suggests that ground shaking intensity decreases as the distance from the seismic source increases, corroborating earlier research^3,4,24^. The study also uncovers a slight positive correlation between PGA and moment magnitude. Furthermore, it is observed that softer soils register higher PGA compared to harder soils. These insights highlight the importance of considering both epicentral distance and moment magnitude for a thorough assessment of seismic impacts on ground motion and structural integrity. This aids in the development of more effective seismic hazard assessments and infrastructure resilience strategies.

## Conclusion

This study investigates the application of Artificial Neural Networks (ANN) for developing Ground Motion Prediction Equations (GMPE) using PEER NGA data. The findings demonstrate that ANNs provide a robust and effective alternative to traditional empirical methods for predicting ground motion, highlighting their potential in seismic hazard assessments and earthquake engineering. Key conclusions include:

- The ANN model exhibits high predictive accuracy, proving to be a valuable tool for estimating ground motion, which is critical for designing safer structures in earthquake-prone regions.
- Machine learning techniques are crucial for analyzing complex, nonlinear patterns in seismic data, thereby improving the reliability of seismic hazard assessments.
- Optimization insights from the study point to areas with potential seismic risks, helping to prioritize safety measures.
- There is substantial potential for further development and application of ANN models in earthquake prediction, which could enhance preparedness and mitigation strategies against earthquake impacts.
- The study encourages ongoing research using machine learning in seismology, emphasizing the significance of advanced data-driven methods for a deeper understanding of ground motion behavior.
- Although the ANN model includes key seismic parameters, future iterations could incorporate additional factors, such as directivity effects, dip angles, and hypocentral distances, to better capture complex seismic phenomena.
- The model’s calibration for specific seismic regions may reduce its broader applicability. Expanding the dataset to include high-resolution, multi-regional seismic data would enhance the model’s robustness and generalizability, making it adaptable to various tectonic settings.

This research sets the stage for more sophisticated, accurate, and practical approaches to understanding and managing seismic risks.

## Data Availability

The data that support the findings of this study are available on request from the corresponding authors.
